# Glucocorticosteroids and bronchopulmonary dysplasia : is epigenetics the missing link?

**DOI:** 10.1038/s41390-024-03203-6

**Published:** 2024-04-16

**Authors:** Olivier Baud, Elodie Zana-Taieb, Daniel Vaiman

**Affiliations:** 1 Division of Neonatology and Pediatric Intensive Care, Children’s University Hospital of Geneva and University of Geneva, Geneva, Switzerland; 2 Inserm U1141, University Paris-Cité, Paris, France; 3 Department of Neonatal Medicine of Port-Royal, Cochin Hospital, FHU PREMA, AP-HP Centre-Université Paris Cité, Paris, France; 4 Institut Cochin, U1016 INSERM, CNRS UMR8104, Faculté de Paris, 24 Rue du Faubourg St Jacques, Paris, 75014 France

Bronchopulmonary dysplasia (BPD) is the most prevalent serious complication developed by infants delivered extremely preterm and its severity was found to proportionally worsen their neurodevelopmental impairment.^1^ Despite recent advances in preclinical and clinical research, understanding its pathogenesis remains unclear, attributed to a complex combination of genetic, prenatal, and postnatal interconnected factors.^2^ Historically, BPD was characterized by lung damage due to aggressive ventilation and supplemental oxygen exposure, referred to as “old” BPD. However, a new pattern termed “new” BPD has emerged,^3^ characterized by altered lung growth and increased elastic tissue without the classic lung damage seen in “old” BPD but still leading to long-term respiratory problems including rehospitalization due to high susceptibility to viral infections. This new concept of disruption in lung development instead of predominant external damage resulting in chronic lung disease has brought genetic predisposition and epigenetic modifications back to the forefront as important players in the BPD pathophysiology.

Hence, recent research, including genome-wide association studies and transcriptomic analyses, has identified potential genetic and epigenetic factors contributing to BPD susceptibility and pathogenesis.^4^ In particular, how DNA methylation analysis of cord blood cells can provide insights into BPD pathogenesis has been reported,^5^ despite challenges related to the dynamic nature of fetal hematopoietic development. Epigenetics involves modifications of DNA bases and histone proteins, as well as noncoding-RNA-based mechanisms regulating host gene expression. While the clinical phenotype of BPD is extremely variable, new technologies have identified alterations of epigenetic regulation involved in lung development and injury, potentially interfering with the influence of airway microbiome.^6^ This rapidly changing landscape suggests that epigenetics-based prognosis and therapeutic strategies may be the future of neonatal pulmonary medicine.

Glucocorticoid hormones, key effectors of stress response, have been associated with changes in epigenetic programming during fetal development.^7^ Maternal antenatal glucocorticosteroids (GC) therapy is considered to be the last major advance in the antenatal management of fetuses at risk of preterm birth. It was adopted worldwide to prevent neonatal death and neonatal complications following preterm birth, including respiratory distress syndrome, necrotizing enterocolitis and severe intraventricular hemorrhage.^8^ However, uncertainty persist regarding its effect on BPD, in particular due to the competing risk with neonatal death in the most high-risk premies.

A recent study published in the Journal explored the relationship between exogenous GC exposure, BPD and stress response system, focusing on epigenetic changes in genes associated with the hypothalamic-pituitary-adrenal (HPA) axis.^9^ The main question was whether specific DNA methylation of HPA axis genes would be associated with BPD severity or with antenatal GC exposure. Blood spot and buccal swabs samples were collected at birth and at intensive care unit discharge, respectively, in two large prospective cohorts of very preterm infants. DNA was quantified via the EPIC microarray and the authors used a polyepigenetic GC score inversely correlated to GC exposure.

The study found that the GC score was not associated with GC exposure or BPD. However, GC score decreased as BPD severity increased, which is indicative of an increased GC exposure, in particular for postnatal GC treatment. Six genes involved in stress response regulation demonstrated differential DNA methylation with antenatal GC exposure, among them 2 were differentially methylated with BPD severity. Males were found to have greater magnitude of differential DNA methylation compared to females.

Association between differential DNA methylation at certain CpG sites within genes such as FKBP5, CRHR1, HSP90AA1, NR3C1, NR3C2, and POMC and prenatal GC exposure was observed only in buccal tissue collected from older infants (close to term equivalent age), suggesting tissue- or age-specific effects.

Because preterm neonates are subjected to multiple environmental stressors known to influence cortisol levels, intra-uterine environment should be considered as a whole part *in se*. Intrauterine growth restriction alters long-term programming of HPA axis^10^ and tight interaction between cortisol metabolism and fetal growth restriction, representing an adaptive response to intrauterine stress and nutrient deprivation. As an example, umbilical cord cortisol concentrations are significantly higher in infants with disruption in fetal growth compared to neonates with stable intra-uterine growth.^11^ Down-regulation of placental 11 beta-HSD2 activity was also associated with lower birthweight.^12^ Authors appropriately considered intra-uterine growth restriction as a potential confounder in adjusted analyses. However, it would be interesting to include postnatal exposure to developmental care as well. Indeed, developmental care emerged few years ago aiming to adapt environment in order to decrease stress. Skin-to-skin care decrease cortisol level and stress in preterm infant,^13^ and studies both in rodents as in humans, showed that maternal caregiving and psychological status modify DNA methylation of genes encoding for the corticotropin-releasing hormone and NR3C1.^14,15^ Therefore, implementation of development care in the NICU should be considered while exploring epigenetics modification of the HPA axis at hospital discharge.

The study did not find significant associations between BPD severity and the GC score or HPA gene methylation, as initially hypothesized. However, binary classification of GC exposure may mitigate detectable effect of various molecules used or cumulative dose which is of major importance here. In addition, looking at blood sample or buccal swabs should also be considered as indirect surrogate of DNA methylation of lung tissue where abnormal biological/inflammatory processes occur in neonates developing BPD. Finally, it acknowledged the possibility that BPD may influence the methylation of many other genes and biological pathways unfortunately not examined in this study. Hence, the main focus of this study upon a limited gene subset, surmising that the HPA axis will be a major sensor of the BPD, is a another limitation. Fortunately, the data now available from the large cohort studied by the authors will allow performing further investigations of interest using the same dataset. One will be the genome-wide analysis of the methylation variations in an agnostic manner. Besides, windows of consecutive CpG might be considered in order to identify Differentially Methylated Regions instead of individual CpGs. These alterations on a given region, even mild, could relate to an actual transcriptional effect on the gene inside which these alterations are found. Another research track would be to analyse the cell composition from the buccal swab as well as from the neonatal blood spots using deconvolution algorithms on methylation, recently shown to be able to divide the blood cells into the major immune cell types, and the buccal cells into immune cells, epithelial cells and fibroblasts.^16^ This could lead to a refined study of the DNA methylation alterations and to insights into possible variations in the cell proportion according to the individual status.

Overall and despite some limitations, this study provides novel insights into the epigenetic impacts of GC treatment and BPD on the HPA axis genes. Further research, including ones using the rich dataset produced by the authors is warranted to confirm and explore these findings, especially using untargeted epigenome-wide approaches to identify additional genes and pathways affected by BPD and its treatments.
